# The probability of fusions joining sex chromosomes and autosomes

**DOI:** 10.1098/rsbl.2020.0648

**Published:** 2020-11-25

**Authors:** Nathan W. Anderson, Carl E. Hjelmen, Heath Blackmon

**Affiliations:** 1 Department of Biology, Texas A&M University, College Station, TX 77843, USA; 2 Department of Integrative Biology, University of Wisconsin, Madison, WI 53706, USA

**Keywords:** sexual antagonism, chromosome fusion, sex determination systems, chromosome number

## Abstract

Chromosome fusion and fission are primary mechanisms of karyotype evolution. In particular, the fusion of a sex chromosome and an autosome has been proposed as a mechanism to resolve intralocus sexual antagonism. If sexual antagonism is common throughout the genome, we should expect to see an excess of fusions that join sex chromosomes and autosomes. Here, we present a null model that provides the probability of a sex chromosome autosome fusion, assuming all chromosomes have an equal probability of being involved in a fusion. This closed-form expression is applicable to both male and female heterogametic sex chromosome systems and can accommodate unequal proportions of fusions originating in males and females. We find that over 25% of all chromosomal fusions are expected to join a sex chromosome and an autosome whenever the diploid autosome count is fewer than 16, regardless of the sex chromosome system. We also demonstrate the utility of our model by analysing two contrasting empirical datasets: one from *Drosophila* and one from the jumping spider genus *Habronattus*. We find that in the case of *Habronattus*, there is a significant excess of sex chromosome autosome fusions but that in *Drosophila* there are far fewer sex chromosome autosome fusions than would be expected under our null model.

## 1. Introduction

The fusion and fission of chromosomes are two of the primary mechanisms that restructure the genome into discrete chromosomes [1]. Fusions and fissions can be selectively favoured in genome restructuring because they modify linkage among loci [2,3]. In particular, the fusion of a sex chromosome and an autosome (SA-fusion) has been proposed to resolve sexual antagonism (when an allele is beneficial for one sex and deleterious for the other) [4]. Linking sexually antagonistic alleles to sex chromosomes can increase the average fitness of both sexes. Therefore, SA fusions are predicted to be more common than fusions joining two autosomes (AA-fusions) [5]. For example, an apparent surplus in X chromosome autosome fusions in the jumping spider genus, *Habronattus*, is hypothesized to result from a mechanism of isolating male-beneficial sexually antagonistic alleles on the neo-Y chromosome [6]. However, most evidence for sexually antagonistic variation comes from within species. For instance, empirical studies in fish, human and flies have found evidence for segregating sexually antagonistic variation (variation with opposite fitness effects in males and females) [7–9]. Furthermore, young sex chromosomes (originating through fusion, translocation or turnover) exhibit signs consistent with the resolution of sexual antagonism [10–12]. However, there remains significant debate on the ubiquity of sexually antagonistic variation and its potential role in genome evolution [13–15]. A strong measure of the frequency of significant sexually antagonistic variation across genomes would be an excess of SA-fusions relative to AA-fusions across large clades. We derive equations describing the probability of each type of fusion necessary to perform such a test and illustrate two approaches to using these equations with empirical datasets. This approach will provide a method to quantitatively analyse the balance of these two types of fusions in the many groups with a well-documented history of fusion between sex chromosomes and autosomes [3,16–18].

## 2. The model

The probability of SA-fusions is a function of the sex chromosome system and the number of autosomes in the genome. To facilitate tests of the balance between SA-fusions and AA-fusions, we have derived a closed-form expression of the probability of a SA-fusion under a null model where any chromosome is equally likely to fuse with any other non-homologous chromosome. Our result is applicable to XO, XY and multi-XY (e.g. X_1_X_2_Y or X_1_X_2_X_3_Y_1_Y_2_) sex chromosome systems and, with slight modification, to ZW and UV systems (reviewed in [19]). We ignore fusions among homologous chromosomes, including fusions that join an X and Y chromosome, because this would lead to unbalanced gametes during meiosis and, presumably, these would be non-viable.

When any two chromosomes fuse, there are three possibilities. The two chromosomes could both be autosomes (AA-fusion), they could both be sex chromosomes (SS-fusion), or one could be a sex chromosome and the other an autosome (SA-fusion). We denote our three possibilities as events AA, SS and SA, respectively. Given that a fusion has occurred, we are interested in the probability that it is an SA-fusion, which can be found by calculating the expected proportion of all fusions that do not involve a sex chromosome:

2.1 P(SA)=1−P(AA)−P(SS).

It is quite possible that the sexes may make unequal contributions to the fusions entering a species. These imbalances could stem from common processes such as meiotic drive or mutation rate differences [20]. We include the term $\mu_{d}$, representing the proportion of fusions that occur in females to account for this possibility. We use a subscript *s* and *d* for sire and dam, respectively, when referring to sex-specific values. We present the following expression for the expected proportion of fusions that occur between two sex chromosomes (equation (2.2)) or two autosomes (equation (2.3)):

2.2 $$P(SS)=\mu_{d}\frac{4X_{s}(X_{s}-1)}{D_{d}(D_{d}-2)}+(1-\mu_{d})\left[\frac{X_{s}(X_{s}-1)}{D_{s}(D_{s}+X_{s}-1)}+\frac{Y(Y-1)}{D_{s}(D_{s}+Y-1)}\right]$$

and

2.3 $$P(AA)=\mu_{d}\frac{D_{a}(D_{a}-2)}{D_{d}(D_{d}-2)}+(1-\mu_{d})\frac{D_{a}(D_{a}-2)}{D_{s}(D_{s}-2)},$$

where $X_{s}$ is X chromosome count in males, $D_{d}$ is female diploid number, $D_{s}\;$is male diploid number, *Y* is Y chromosome count in males and $D_{a}$ is diploid autosome count.

Each fraction represents the probability of two types of chromosomes fusing using a counting argument. For instance, the fraction $D_{a}(D_{a}-2)/D_{s}(D_{s}-2)$ in equation (2.3) represents the probability of a fusion joining two autosomes in a male.

Substituting equations (2.2) and (2.3) into equation (2.1) yields:

2.4 $$\begin{array}{c} P(SA)=1-\mu_{d}\frac{D_{a}(D_{a}-2)+4X_{s}(X_{s}-1)}{D_{d}(D_{d}-2)}\;\\ \;\;\;-(1-\mu_{d})[\frac{D_{a}(D_{a}-2)}{D_{s}(D_{s}-2)}+\frac{X_{s}(X_{s}-1)}{D_{s}(D_{s}+X_{s}-1)}\;\\ \;\;\;\;+\frac{Y(Y-1)}{D_{s}(D_{s}+Y-1)}]\; \end{array}.$$

**Figure 1.**
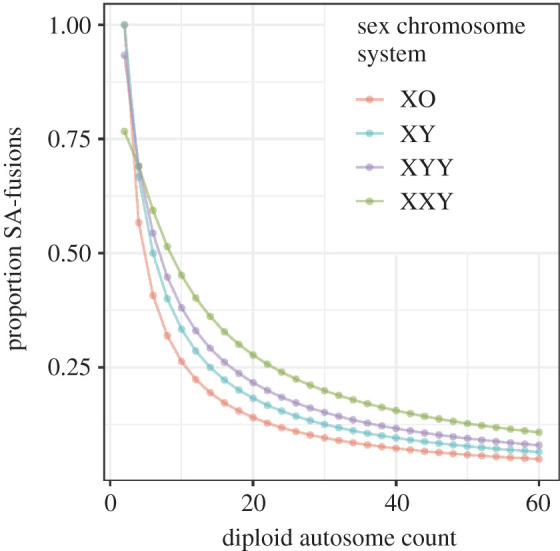
Probability of a random fusion joining a sex chromosome and autosome. On the vertical axis, we plot the proportion SA-fusions, while on the horizontal axis, we plot the diploid autosome count. Sex chromosome system is indicated by colour.

This equation allows us to calculate the expected proportion of SA-fusions for any XY sex chromosome system with any number of autosomes (figure 1). Equations (2.2)–(2.4) have six parameters: $\mu_{d}$, $X_{s}$, $D_{a}$, *Y*, $D_{d}$ and $D_{s}$. We avoid the parameter $X_{d}$, the number of X chromosomes in females, by noting $X_{d}=2X_{s}$. This formulation can be converted for use in ZW sex chromosome systems by exchanging $D_{d}$ and $D_{s}$, replacing $X_{s}$ with $Z_{d}$, replacing *Y* with *W* and replacing $\mu_{d}$ with $\mu_{s}$. Additionally, setting $\mu_{d}=0$ (because there are no homogametic diploid individuals) and replacing both $X_{s}$ and Y for V generate equations that are accurate for UV sex chromosome systems, in cases where there are an equal number of U and V chromosomes (detailed derivation in electronic supplementary material). We have provided R functions that calculate *P*(*SA*), *P*(*SS*) and *P*(*AA*) in the R package evobiR [21]. The model that we have developed will allow for the identification of clades that exhibit significant deviations from a neutral expectation that all fusions are equally likely.

## 3. Empirical applications

To demonstrate the utility of our approach, we apply our equations in two empirical systems. The first is the jumping spiders genus *Habronattus* that has been suggested to show a large excess of SA-fusions [6,22], and the second is *Drosophila*, which has served as a model system for much of our understanding of sex chromosomes [5,12,23].

### (a) Habronattus

In a recent study of *Habronattus* jumping spiders, the large disparity between the number of SA-fusions (8–15) and AA-fusions (1) and SS-fusions (1) in a system with approximately 26 autosomes is presented as evidence that SA-fusions are being favoured [6]. The intuition that this imbalance is unlikely can be rigorously tested with our null model that the distribution of fusions is determined by chromosome number and sex chromosome system. Using equations (2.2)–(2.4) and a multinomial distribution, we are able to calculate the exact empirical *p*-value of having observed eight or more SA-fusions out of a total of 10 fusions. We assume an XXO sex chromosome system and a diploid autosome count of 26 (this karyotype was the most common in the ancestral state estimation performed in the study).

$$\begin{array}{r} P(8\;\mathrm{SA}-\mathrm{fusions}\;\mathrm{out}\;\mathrm{of}\;10)=\\ \;\sum_{i=8}^{10}\sum_{\;j=0}^{10-i}\frac{10!}{i!\cdot j!\cdot (10-i-j)!}P{\left(SA\right)}^{i}\cdot P{\left(AA\right)}^{j}\cdot P{\left(SS\right)}^{10-i-j}<10^{-5}. \end{array}$$

This confirms that *Habronattus* spiders do in fact have an excess of SA-fusions. In this example, we calculated the expected proportion of the different types of fusions based on the most common karyotype inferred in the clade. However, we envision the primary use of equation (2.4) will be to calculate the expected proportion of SA-fusions across large clades that have many changes in chromosome number and sex chromosome system. We illustrate this approach below.

### (b) Drosophila

**Figure 2.**
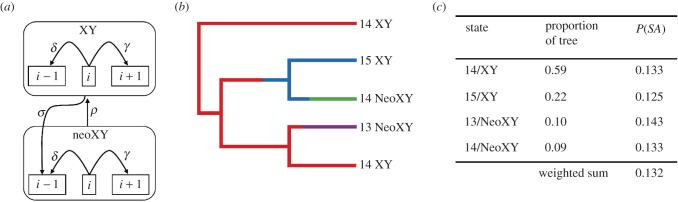
Estimations across a phylogeny (*a*) Markov model for the evolution of karyotypes in *Drosophila*. A lineage will have i chromosomes and either an XY or neoXY sex chromosome system, and can make four possible transitions: δ, the AA-fusion; γ, the fission of an autosome; σ, SA-fusion; and ρ the transition from neoXY to XY. (*b*) A stochastic map showing one possible history for these traits. (*c*) Calculation of the weighted expected P(SA) for the clade as a whole.

We employ a biologically realistic Markov model of karyotype evolution (figure 2*a*) and leverage stochastic mappings (figure 2*b*) [24,25] to extract the proportion of time that lineages in a clade spent with each possible chromosome number and sex chromosome system. These proportions used in conjunction with equation (2.4) provide a weighted sum that describes the expected proportion of SA-fusions (figure 2*c*). The resulting expected value can then be compared to the observed proportion of SA-fusions inferred from stochastic mappings. An additional advantage of this approach is that it can incorporate uncertainty in both ancestral state reconstructions and phylogenetic history.

We used a dated ultrametric phylogeny, chromosome number and sex chromosome system data from recent studies [26,27]. This yielded a dataset consisting of 120 species with a diploid number ranging from 6 to 12. The sex chromosome system of eleven of the species was neoXY (term used to describe a karyotype where a sex chromosome and autosome have fused forming a larger ‘new' sex chromosome) while the remainder were XY. Using the R package phytools [25], we performed 1000 stochastic mappings using the make.simmap function and extracted the time spent in each state along the phylogeny and the number of each type of fusion using the describe.simmap function. Stochastic mapping was accomplished using a transition matrix that matches the Markov model presented in figure 2*a*. We performed a number of preliminary analyses where we assessed the impact of (i) the prior placed on the root of the tree and (ii) the inclusion of ρ (figure 2*a*). We found that our results were qualitatively the same under all evaluated conditions. The results we present are based on fixing the root of the tree with a diploid number of 12 and an XY sex chromosome system, and including the parameter ρ in the model. The prior on the root of the tree is supported by comparative genomic studies [27]. The inclusion of ρ is based on our concern that some species may harbour an undocumented neoXY. We found that including ρ in the model elevated our estimate of the proportion of SA fusions but not sufficiently to change the interpretation of the results.

Across our 1000 stochastic mappings, we find that the average number of SA-fusions observed is 4.49 and that this equates to a proportion of 0.155 (credible interval 0.12–0.22). Using our formula as described above we also calculated the expected proportion of SA-fusions. The mean expected SA-fusion proportion was 0.43 (credible interval 0.42–0.44). Comparing these distributions (electronic supplementary material, figure S2), we find that they have zero overlap and that the empirical dataset shows far fewer SA fusions than would be expected by chance.

## 4. Discussion

The need for a quantitative null model of the probability of SA-fusions is illustrated by examining the expected probability of SA-fusions across a range of observed chromosome numbers and sex chromosome systems. In figure 1, we show that when the autosome number is small, a large proportion of fusions are expected to be SA-fusions even under a null model that assumes they are not selectively favoured. In fact, for an XY sex chromosome system, the probability of a given fusion being an SA-fusion does not drop below 25% until the diploid autosome count is greater than or equal to 16. In systems with XXY sex chromosomes, the case is even more extreme. The probability of SA-fusion does not drop below 25% until the diploid autosome count is greater than 22. Therefore, evaluating the proportion of SA-fusions and determining whether there is evidence for positive selection for these fusions can only be accomplished in light of a quantitative null model that takes account of chromosome number and sex chromosome system.

Previous work examining SA-fusions in *Drosophila* has largely focused on the balance between fusions of an autosome with the X versus the Y [23]. Much of this work was done prior to the development of modern comparative approaches and could not fully incorporate the evolution of chromosome number over the history of *Drosophila*. In our analysis, we asked how the number of AA-fusions compare with the number of SA-fusions. Our results show that *Drosophila* have far fewer SA-fusions than would be expected if all fusions were equal.

The scarcity of SA-fusions that we document suggests that in *Drosophila* SA-fusions are more likely to have deleterious effects than fusions that join two autosomes. One possible explanation for apparent selection against SA-fusions in *Drosophila* may lie in the joint action of genome structure and a lack of recombination in males (achiasmatic meiosis). In species with achiasmatic meiosis, when an SA-fusion occurs, the entire Y chromosome is immediately subject to population genetic forces (e.g. Muller's ratchet) that lead to the loss of functional genes [28]. *Drosophila* has relatively few chromosomes such that each chromosome carries many genes (in *D. melanogaster* 43% of all genes are on autosome 3) [29]. Therefore, while an SA-fusion may initially provide a fitness benefit, the fitness benefit may quickly decay owing to the ‘target size' for deleterious mutations on the Y chromosome precluding the fusion's fixation. Testing this hypothesis across multiple clades with variation in meiotic mechanisms should reveal whether this is a general pattern.

We have developed a flexible equation used to calculate the probability of SA-fusions under most sex chromosome systems. This model will allow for quantitative analyses of fusions across large clades and provide a way to test the long-standing hypothesis that SA-fusions are selectively favoured for their ability to resolve sexual antagonism. In some clades where chromosome number is high (e.g. Lepidoptera and Isoptera), our model shows that SA-fusions should be rare. In these cases, several SA-fusions within a clade may well suggest that these fusions are selectively favoured. However, this model also shows that for clades with very few chromosomes (e.g. Diptera and Hemiptera), we should expect many SA-fusions even if they are not selectively favoured. Therefore, SA-fusions should only be considered as evidence for sexual antagonism when they occur at a higher rate than expected for the chromosome numbers and sex chromosome systems that have been present during the evolution of a clade.

## Supplementary Material

Supplementary Material

## Data accessibility

Code and data to reproduce this study are available via GitHub: https://github.com/coleoguy/sex-autosome-fusion (doi:10.5281/zenodo.4118382) [30].
