# Supplementary material for: The probability of fusions joining sex chromosomes and autosomes
Source: Biol Lett. 2020 Nov 25;16(11):20200648. doi: 10.1098/rsbl.2020.0648 (PMC7728677; doi:10.1098/rsbl.2020.0648)
Supplement: Supplementary Material [file rsbl20200648supp1.docx]

**The Probability of Fusions Joining Sex Chromosomes and Autosomes**

Nathan W. Anderson^1,2^, Carl E. Hjelmen^1^, and Heath Blackmon^1,3^

^1^Department of Biology; Texas A&M University; College Station, TX 77843, USA

^2^Department of Integrative Biology; University of Wisconsin; Madison WI 53706, USA

^3^Author for correspondence: HB, [*coleoguy@gmail.com*](mailto:coleoguy@gmail.com)

# Mechanisms of genome restructuring

While we have focused this work on fusions, we recognize that many types of chromosomal rearrangements, such as inversions, fusions, fissions, and translocations, can all dramatically reshape the genomic landscape of species, their sexes, and populations. In fact, in a study by Bush in 1977 it was found that speciation was strongly positively correlated with the rate of chromosomal evolution in mammals [1]. This correlation was further supported in a recent study of chromosomal fusions and fissions in Lepidoptera [2]. Since early studies in *Drosophila* by Sturtevant, inversions have been shown to segregate among populations and provide evidence of population structure [3-5]. While some of these inversions are neutral and do not provide a known benefit, other inversions link alleles beneficial to survival due to the biotic and abiotic environment a population experiences, resulting in local adaptation [reviewed in 6, 7-9] and eventual speciation, as seen across introgression studies in *Drosophila* and mosquitoes [10-13]. Outside of their role in replenishing the pseudoautosomal region of sex chromosomes and formation of neo-sex chromosomes [14-16], fusions, like inversions, reduce recombination between loci, by bringing genes which were previously on separate chromosomes together, providing a similar role in local adaptation as described above [17-19]. In fact, the models developed by Guerroro and Kirkpatrick for the role of fusions in local adaptation also apply to more specific types of fusions (end-to-end, tandem, fusions of holocentric chromosomes) as well as to other types of chromosomal rearrangements, such as Robertsonian translocations and reciprocal translocations [17]. Translocations involving sex chromosomes, however, can dramatically reshape sex linkage more than inversions as seen in frog, *Rana temporaria* [20, 21]. Just as the structure of chromosomes is not static the position of individual genes is also labile. In particular there is abundant evidence that gene duplication can produce both tandem copies closely linked to the original gene copy as well as duplicates on entirely different chromosomes[22, 23]. These distant duplicates often show a pattern that suggests that movement onto or off of sex chromosomes is beneficial [24-27]

# Model derivation

We wish to derive an expression for the probability of a fusion between a sex chromosome and an autosome assuming that any chromosome is equally likely to be involved in the fusion with the exception of fusions between homologous chromosomes, and fusions between an X and a Y (because these would lead to unbalanced gametes during meiosis and would presumably be nonviable). Directly calculating the probability of a sex autosome fusion proves to be challenging and can be avoided by taking advantage of the compliment rule:

$P\left( SA \right)=1-P(AA)-P\left( SS \right)$ (A1)

Note that given a fusion occurs, the only three possibilities are that it is between two sex chromosome, two autosomes, or between a sex chromosome and an autosome. It is quite possible that the sexes may make unequal contributions to the fusions entering a species (Pennell et al. 2015). Because of this, we must account for the differing probabilities in males and females. We have added the term $\mu_{d}$, representing the proportion of fusions that occur in females, and partitioned $P(AA)$ and $P\left( SS \right)$ into their sex specific components to account for this possibility. We use a subscript $s$ and $d$ for sire and dam when referring to sex specific values to avoid any confusion stemming from using subscript $m$ and $f$.

$P\left( SS \right)=\mu_{d}P\left( {SS}_{d} \right)+(1-\mu_{d})P({SS}_{s})$ (A2)

$P\left( AA \right)=P\left( \mathrm{AA}_{d} \right)+(1-\mu_{d})P(\mathrm{AA}_{s})$ (A3)

We will find an expression for each of the probabilities. Each term is calculated using a counting argument. These are the classic “marbles in an urn” probability arguments. The probability of choosing a red marble is the proportion of red marbles in the urn, just as the probability of ‘choosing’ an autosome is the number of autosomes available over the total number of chromosomes available to be ‘chosen’. A graphical description of each counting problem is given in figure A1, in the case of a XXYYY sex chromosome system with 6 autosomes, but the generalization follows easily.

$P\left( \mathrm{SS}_{d} \right)=P\left( X \right)P\left( X | X \right)=\frac{2X_{s}(2X_{s}-2)}{D_{d}(D_{d}-2)}$ (A4)

(fig A1, yellow square)

$P\left( \mathrm{SS}_{s} \right)=P\left( X \right)P\left( X | X \right)+P\left( Y \right)P(Y|Y)=\frac{X_{s}(X_{s}-1)}{D_{s}(D_{a}+X_{a}-1)}+\frac{Y(Y-1)}{D_{s}(D_{a}+Y-1)}$ (A5)

(fig A1, purple and green squares)

$P\left( \mathrm{AA}_{d} \right)=P\left( A \right)P\left( A | A \right)=\frac{D_{a}(D_{a}-2)}{D_{d}(D_{d}-2)}$ (A6)

(fig A1, red square)

$P\left( \mathrm{AA}_{s} \right)=P\left( A \right)P\left( A | A \right)=\frac{D_{a}(D_{a}-2)}{D_{s}(D_{s}-2)}$ (A7)

(fig A1, blue square)

Equations [A4]-[A7] can then be substituted into equations [A2] and [A3] yielding the following expressions for the proportion of SS and AA fusions:

$P(SS)=\mu_{d}\frac{4X_{s}(X_{s}-1)}{D_{d}(D_{d}-2)}+(1-\mu_{d})\left[ \frac{X_{s}(X_{s}-1)}{D_{s}(D_{s}+X_{s}-1)}+\frac{Y(Y-1)}{D_{s}(D_{s}+Y-1)} \right]$ (A8)

$P(AA)=\mu_{d}\frac{D_{a}(D_{a}-2)}{D_{d}(D_{d}-2)}+(1-\mu_{d})\frac{D_{a}(D_{a}-2)}{D_{s}(D_{s}-2)}$ (A9)

Substituting equations [A8] and [A9] into equation [A1] gives us our result:

$P(SA)=1-\mu_{d}\frac{D_{a}(D_{a}-2)+4X_{s}(X_{s}-1)}{D_{d}(D_{d}-2)}-(1-\mu_{d})\left[ \frac{D_{a}(D_{a}-2)}{D_{s}(D_{s}-2)}+\frac{X_{s}(X_{s}-1)}{D_{s}(D_{s}+X_{s}-1)}+\frac{Y(Y-1)}{D_{s}(D_{s}+Y-1)} \right]$ (A10)


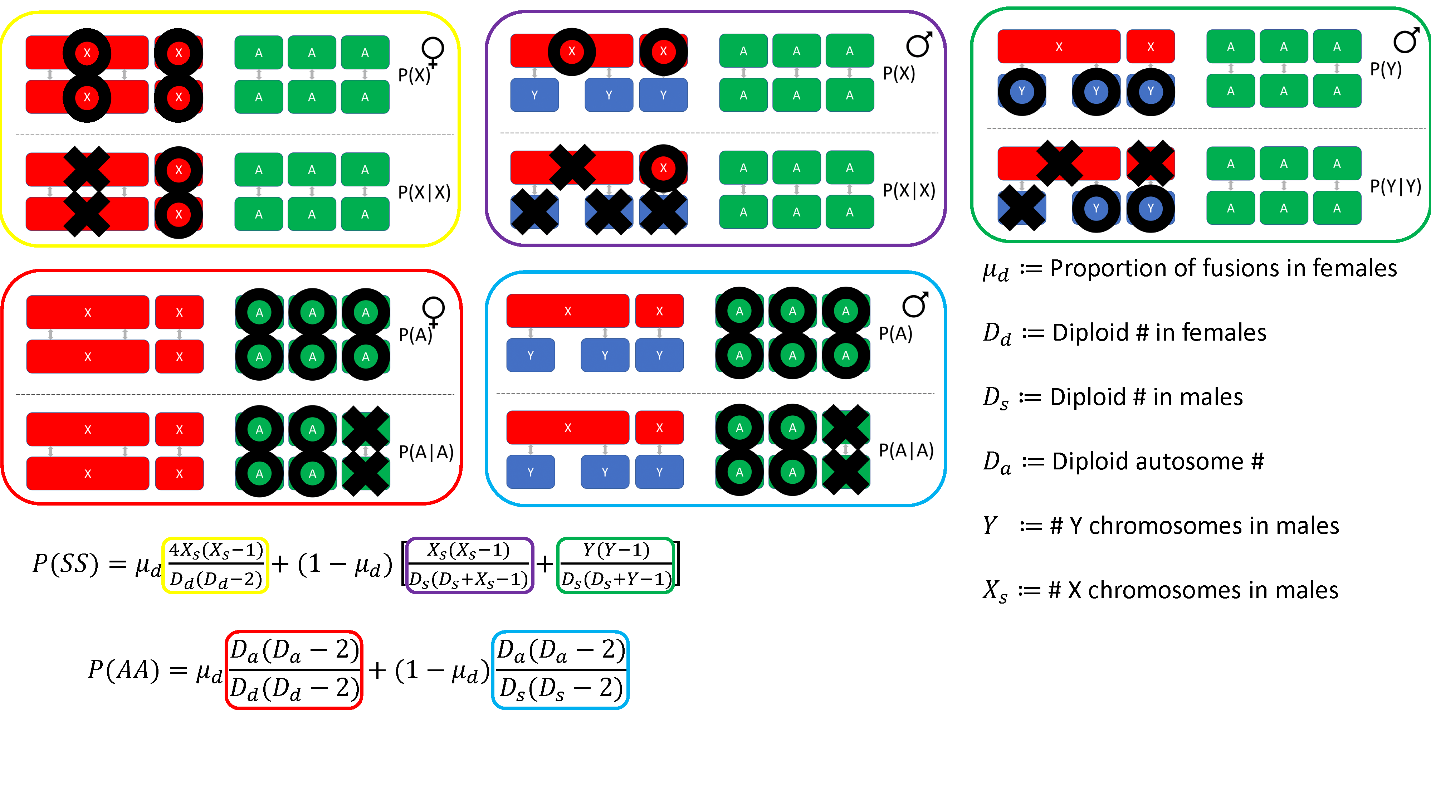


**Figure S1 Graphical representation of the counting argument** used to derive each fraction in the case of an XXYYY system with a diploid autosome count of six. Circles are found on the chromosome we are ‘choosing’, and X’s are placed on chromosomes which are unavailable to be ‘chosen’ due to a previously ‘chosen’ chromosome. The diagrams represent the fraction surrounded by the box of matching color. For instance, the fraction in the red box represents P(A)P(A|A) in females (equation [A6]), the probability of an autosome is first ‘chosen’ to fuse, and a second autosome is ‘chosen’ to fuse with it and is represented by the diagram in the box of the same color. Inside the box, the diagram above the dotted line shows the probability of choosing one of the six autosomes out of all of the chromosomes. The diagram below the line shows the probability of choosing one of the four autosomes remaining available to be chosen after the first autosome had been chosen. The other fractions follow similarly.

# Drosophila analysis


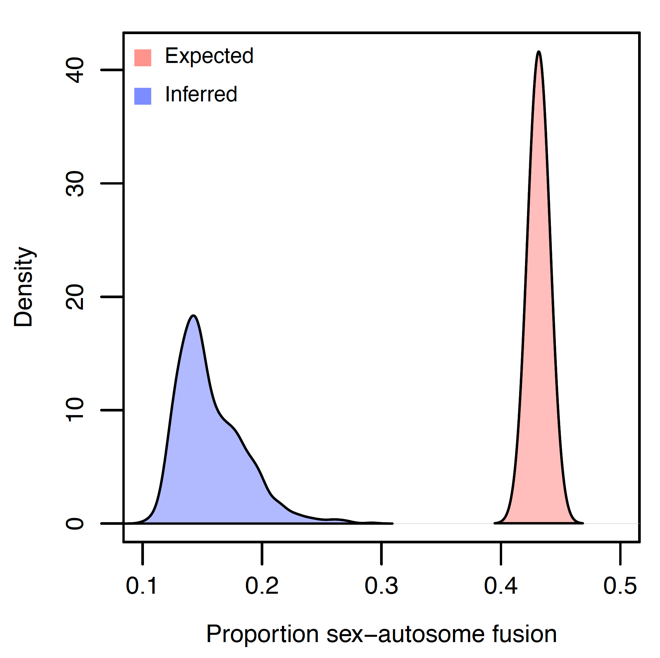


**Figure S2 Observed and expected proportions of fusions joining a sex chromosome and an autosome.** Our results indicate a scarcity of fusions joining sex chromosomes and autosomes. Of the 1000 stochastic mappings evaluated, none of the possible evolutionary histories sampled suggested a proportion of sex chromosome autosome fusions that would be expected under the null model. This is strong evidence that sex autosome fusions are either more deleterious than autosome autosome fusions or that de novo fusions joining a sex chromosome and an autosome occur more infrequently than do fusions joining autosomes.

[1] Bush, G.L., Case, S., Wilson, A. & Patton, J. 1977 Rapid speciation and chromosomal evolution in mammals. *Proceedings of the National Academy of Sciences* **74**, 3942-3946.

[2] de Vos, J.M., Augustijnen, H., Bätscher, L. & Lucek, K. 2020 Speciation through chromosomal fusion and fission in Lepidoptera. *Philosophical Transactions of the Royal Society B* **375**, 20190539.

[3] Sturtevant, A.H. 1917 Genetic factors affecting the strength of linkage in Drosophila. *Proceedings of the National Academy of Sciences of the United States of America* **3**, 555.

[4] Dobzhansky, T. & Sturtevant, A.H. 1938 Inversions in the chromosomes of Drosophila pseudoobscura. *Genetics* **23**, 28.

[5] Sturtevant, A.H. 1921 A case of rearrangement of genes in Drosophila. *Proceedings of the National Academy of Sciences of the United States of America* **7**, 235.

[6] Kirkpatrick, M. & Barton, N. 2006 Chromosome inversions, local adaptation and speciation. *Genetics* **173**, 419-434.

[7] Kirkpatrick, M. 2010 How and why chromosome inversions evolve. *PLoS Biol* **8**, e1000501.

[8] Savolainen, O., Lascoux, M. & Merilä, J. 2013 Ecological genomics of local adaptation. *Nature Reviews Genetics* **14**, 807-820.

[9] Christmas, M.J., Wallberg, A., Bunikis, I., Olsson, A., Wallerman, O. & Webster, M.T. 2019 Chromosomal inversions associated with environmental adaptation in honeybees. *Molecular ecology* **28**, 1358-1374.

[10] Lohse, K., Clarke, M., Ritchie, M.G. & Etges, W.J. 2015 Genome‐wide tests for introgression between cactophilic Drosophila implicate a role of inversions during speciation. *Evolution* **69**, 1178-1190.

[11] Powell, J., Petrarca, V., Della Torre, A., Caccone, A. & Coluzzi, M. 1999 Population structure, speciation, and introgression in the Anopheles gambiae complex. *Parassitologia* **41**, 101-113.

[12] Della Torre, A., Merzagora, L., Powell, J. & Coluzzi, M. 1997 Selective introgression of paracentric inversions between two sibling species of the Anopheles gambiae complex. *Genetics* **146**, 239-244.

[13] Kulathinal, R.J., Stevison, L.S. & Noor, M.A. 2009 The genomics of speciation in Drosophila: diversity, divergence, and introgression estimated using low-coverage genome sequencing. *PLoS Genet* **5**, e1000550.

[14] Blackmon, H. & Demuth, J.P. 2015 The fragile Y hypothesis: Y chromosome aneuploidy as a selective pressure in sex chromosome and meiotic mechanism evolution. *BioEssays* **37**, 942-950. (doi:doi:10.1002/bies.201500040).

[15] Bachtrog, D. 2008 The temporal dynamics of processes underlying Y chromosome degeneration. *Genetics* **179**, 1513-1525. (doi:<https://doi.org/10.1534/genetics.107.084012>).

[16] Satomura, K. & Tamura, K. 2016 Ancient male recombination shaped genetic diversity of neo-Y chromosome in Drosophila albomicans. *Molecular Biology and Evolution* **33**, 367-374.

[17] Guerrero, R.F. & Kirkpatrick, M. 2014 Local adaptation and the evolution of chromosome fusions. *Evolution* **68**, 2747-2756.

[18] Wellband, K., Mérot, C., Linnansaari, T., Elliott, J., Curry, R.A. & Bernatchez, L. 2019 Chromosomal fusion and life history‐associated genomic variation contribute to within‐river local adaptation of Atlantic salmon. *Molecular ecology* **28**, 1439-1459.

[19] Tigano, A. & Friesen, V.L. 2016 Genomics of local adaptation with gene flow. *Molecular ecology* **25**, 2144-2164.

[20] Toups, M.A., Rodrigues, N., Perrin, N. & Kirkpatrick, M. 2019 A reciprocal translocation radically reshapes sex‐linked inheritance in the common frog. *Molecular ecology* **28**, 1877-1889.

[21] Scott, M.F. 2019 Causes and consequences of reciprocal translocations on sex chromosomes. *Molecular ecology* **28**, 1863-1865.

[22] Zhang, J. 2003 Evolution by gene duplication: an update. *Trends in ecology & evolution* **18**, 292-298.

[23] Casola, C. 2018 From de novo to “de nono”: the majority of novel protein-coding genes identified with phylostratigraphy are old genes or recent duplicates. *Genome biology and evolution* **10**, 2906-2918.

[24] Dupim, E.G., Goldstein, G., Vanderlinde, T., Vaz, S.C., Krsticevic, F., Bastos, A., Pinhão, T., Torres, M., David, J.R. & Vilela, C.R. 2018 An investigation of Y chromosome incorporations in 400 species of Drosophila and related genera. *PLoS genetics* **14**, e1007770.

[25] Betrán, E., Thornton, K. & Long, M. 2002 Retroposed new genes out of the X in Drosophila. *Genome research* **12**, 1854-1859.

[26] Emerson, J., Kaessmann, H., Betrán, E. & Long, M. 2004 Extensive gene traffic on the mammalian X chromosome. *Science* **303**, 537-540.

[27] Zhang, Y.E., Vibranovski, M.D., Landback, P., Marais, G.A. & Long, M. 2010 Chromosomal redistribution of male-biased genes in mammalian evolution with two bursts of gene gain on the X chromosome. *PLoS Biol* **8**, e1000494.
